# Novel Mutation p.Asp374Val of *SERPINC1* in a Turkish Family with Inherited Antithrombin Deficiency

**DOI:** 10.4274/tjh.galenos.2021.2020.0702

**Published:** 2021-06-01

**Authors:** Deniz Aslan

**Affiliations:** 1 Gazi University Faculty of Medicine, Department of Pediatrics, Division of Hematology, Ankara, Turkey

**Keywords:** p.Asp374Val mutation, SERPINC1, Inherited antithrombin deficiency

## To the Editor,

Antithrombin (AT) is a major inhibitor of blood coagulation. It is a serine protease inhibitor (SERPIN) that degrades thrombin, factor (F) IXa, FXa, FXIa, and FXIIa. It is constantly active, but the presence of heparan sulfate or the administration of heparins will further increase this inactivation [1]. Inherited AT deficiency (ATD) (OMIM #107300) is a rare autosomal dominant disorder associated with an increased risk of venous thromboembolism (VTE), which usually develops in young and middle-aged adults [2].

The AT gene *(SERPINC1)* contains seven exons and six introns [2]. Approximately 400 distinct mutations in *SERPINC1* have been described [3]. ATD is characterized by either a reduced level of circulating protein (quantitative, type I) or by the presence of variant proteins (qualitative, type II) [4]. We herein report a novel mutation, a base pair substitution (c.1121A>T) leading to p.Asp374Val, in *SERPINC1* in a patient with inherited ATD presenting with cerebral sinovenous thrombosis.

The proband was a 16-year-old Turkish male with a family history of deep venous thrombosis (DVT). He was hospitalized after two days of recurrent headache. Diffusion magnetic resonance imaging (MRI) and venography showed thrombosis in the right transverse sinus and right sigmoid sinus. He was diagnosed with cerebral sinovenous theombosis.

**Figure 1:**
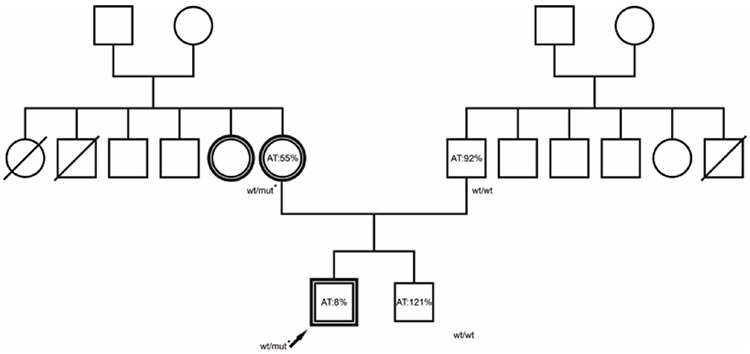
The pedigree of the proband. A double circle or square represents an individual with a definite history of venous thromboembolism. Wt, wild-type; mut*, the p.Asp374Val mutation of *SERPINC1*, the mutation identified in the proband.

He had no acquired risk factors such as obesity, infection, immobility, or trauma. Hematological work-up revealed reduced AT activity (8%; normal range: 80%-120%) (STA^®^-Stachrom^®^ AT III Kit). Factor V Leiden (FVL), prothrombin 20210A mutation, presence of antiphospholipid and anticardiolipin antibodies, and protein C or S deficiency were excluded. Fibrinogen and thrombin time results were within normal limits. His mother and maternal aunt had been treated for unprovoked DVT during their thirties without thorough investigation of the underlying cause. The remaining family members had no history of DVT. Hematological evaluation of the core family revealed a reduced AT level in the mother (Figure 1). The level of AT in the maternal aunt was not available. None of the family members had liver or kidney disease, and there were no other factors contributing to AT reduction.

The patient was successfully treated with subcutaneous enoxaparin at 1.0 mg/kg twice a day (adjusted according to anti-FXa levels), which was continued for 18 months, when full recanalization of the affected sinuses was observed. The AT level gradually increased over the course of therapy and stabilized at around 60%. The two-year follow-up without prophylaxis was uneventful.

**Figure 2:**
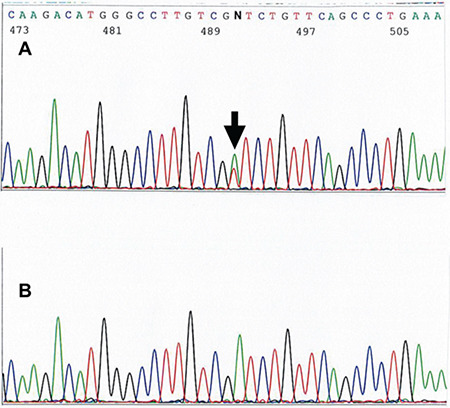
Sequence diagram showing heterozygous c.1121A>T in the proband **(A)** and, for comparison, normal sequencing in the father **(B)**. The position of the mutational base is indicated with an arrow.

After obtaining informed consent, the exons and intron-exon boundaries of *SERPINC1* were studied by direct sequencing [5]. A mutation of p.Asp374Val present in the heterozygous state was found (Figure 2). An in silico study [6] confirmed that this mutation could be detrimental. A search of the published literature revealed that this mutation, named “AT Ankara”, has not been previously reported.

VTE is a multifactorial disease determined by a combination of environmental and genetic risk factors. Inherited ATD is a recognized strong genetic risk factor for VTE [7,8,9]. A meta-analysis of observational studies showed a high risk of first (16-fold) and recurrent (4-fold) VTE in ATD [10].

This novel c.1121A>T, a missense mutation, causes the replacement of a charged amino acid (Asp) with a non-polar one (Val), leading to p.Asp374Val. The change in the amino acid alters the AT protein, leading to a loss of specific inhibitory activity, and increases the risk of thrombosis at a young age. Another mutation at this position (c.1121A>G), leading to p.D374G associated with type II deficiency, has been previously described [11]. Different amino acid changes at the same position, causing distinct clinical phenotypes, have been described in other diseases [12]. The clinical severity of VTE due to AT Ankara and the risk of arterial thrombosis development, if any, are not yet clear; accumulating reports of further cases carrying the same mutation will clarify these points.

Our patient, with a young age and an active lifestyle, was followed without prophylaxis since he had no predisposing factors and the other affected family members had no recurrence. Furthermore, no recurrence was observed in the previous patient with a mutation in the same position despite additional risk factors of arterial hypertension and heterozygous state of FVL [11]. However, management of inherited ATD should be determined on an individual basis and prophylaxis should be considered in cases with predisposing factors.

In conclusion, we have presented herein a novel mutation in *SERPINC1* as the genetic cause of VTE in a Turkish family.

## References

[1] Sarangi SN, Acharya SA (2016.). Disorders of Coagulation. In: Lanzkowsky P, Lipton JM, Fish JD (eds). Lanzkowsky’s Manual of Pediatric Hematology and Oncology. New York, Academic Press.

[2] Olds RJ, Lane DA, Chowdhury V, De Stefano V, Leone G, Thein SL (1993). Complete nucleotide sequence of the antithrombin gene: evidence for homologous recombination causing thrombophilia. Biochemistry.

[3] Zhang H, Liu S, Luo S, Jin Y, Yang L, Xie H, Pan J, Wang M (2020). Two novel mutations cause hereditary antithrombin deficiency in a Chinese family. Acta Haematol.

[4] Patnaik MM, Moll S (2008). Inherited antithrombin deficiency: a review. Haemophilia.

[5] Picard V, Dautzenberg MD, Villoutreix BO, Orliaguet G, Alhenc-Gelas M, Aiach M (2003). Antithrombin Phe229Leu: a new homozygous variant leading to spontaneous antithrombin polymerization in vivo associated with severe childhood thrombosis. Blood.

[6] Richards S, Aziz N, Bale S, Bick D, Das S, Gastier-Foster J, Grody WW, Hegde M, Lyon E, Spector E, Voelkerding K, Rehm HL;, ACMG Laboratory Quality Assurance Committee (2015). Standards and guidelines for the interpretation of sequence variants: a joint consensus recommendation of the American College of Medical Genetics and Genomics and the Association for Molecular Pathology. Genet Med.

[7] Carrell RW, Lomas DA (2002). Alpha1-antitrypsin deficiency--a model for conformational diseases. N Engl J Med.

[8] Sarper N, Orlando C, Demirsoy U, Gelen SA, Jochmans K (2014). Homozygous antithrombin deficiency in adolescents presenting with lower extremity thrombosis and renal complications: two case reports from Turkey. J Pediatr Hematol Oncol.

[9] de la Morena-Barrio B, Orlando C, de la Morena-Barrio ME, Vicente V, Jochmans K, Corral J (2019). Incidence and features of thrombosis in children with inherited antithrombin deficiency. Haematologica.

[10] Di Minno MN, Ambrosino P, Ageno W, Rosendaal F, Di Minno G, Dentali F (2015). Natural anticoagulants deficiency and the risk of venous thromboembolism: a meta-analysis of observational studies. Thromb Res.

[11] Castaldo G, Cerbone AM, Guida A, Tandurella I, Ingino R, Tufano A, Ceglia C, Di Minno MN, Ruocco AL, Di Minno G (2012). Molecular analysis and genotype-phenotype correlation in patients with antithrombin deficiency from Southern Italy. Thromb Haemost.

[12] Bradley JF, Collins DL, Schimke RN, Parrott HN, Rothberg PG (1999). Two distinct phenotypes caused by two different missense mutations in the same codon of the VHL gene. Am J Med Genet.

